# Mitochondrial Genomes of Kinorhyncha: *trnM* Duplication and New Gene Orders within Animals

**DOI:** 10.1371/journal.pone.0165072

**Published:** 2016-10-18

**Authors:** Olga V. Popova, Kirill V. Mikhailov, Mikhail A. Nikitin, Maria D. Logacheva, Aleksey A. Penin, Maria S. Muntyan, Olga S. Kedrova, Nikolai B. Petrov, Yuri V. Panchin, Vladimir V. Aleoshin

**Affiliations:** 1 Faculty of Bioengineering and Bioinformatics, Lomonosov Moscow State University, Leninskie Gory, 1, build. 73, Moscow 119991, Russian Federation; 2 Belozersky Institute for Physico-Chemical Biology, Lomonosov Moscow State University, Leninskie Gory, 1, build. 40, Moscow 119991, Russian Federation; 3 Institute for Information Transmission Problems, Russian Academy of Sciences, Bolshoy Karetny per. 19, build. 1, Moscow 127994, Russian Federation; 4 Extreme Biology Laboratory, Institute of Fundamental Medicine and Biology, Kazan Federal University, 18 Kremlyovskaya str., Kazan 420008, Russia Federation; 5 Faculty of Biology, Lomonosov Moscow State University, Leninskie Gory, 1, build. 12, Moscow 119991, Russian Federation; Sichuan University, CHINA

## Abstract

Many features of mitochondrial genomes of animals, such as patterns of gene arrangement, nucleotide content and substitution rate variation are extensively used in evolutionary and phylogenetic studies. Nearly 6,000 mitochondrial genomes of animals have already been sequenced, covering the majority of animal phyla. One of the groups that escaped mitogenome sequencing is phylum Kinorhyncha—an isolated taxon of microscopic worm-like ecdysozoans. The kinorhynchs are thought to be one of the early-branching lineages of Ecdysozoa, and their mitochondrial genomes may be important for resolving evolutionary relations between major animal taxa. Here we present the results of sequencing and analysis of mitochondrial genomes from two members of Kinorhyncha, *Echinoderes svetlanae* (Cyclorhagida) and *Pycnophyes kielensis* (Allomalorhagida). Their mitochondrial genomes are circular molecules approximately 15 Kbp in size. The kinorhynch mitochondrial gene sequences are highly divergent, which precludes accurate phylogenetic inference. The mitogenomes of both species encode a typical metazoan complement of 37 genes, which are all positioned on the major strand, but the gene order is distinct and unique among Ecdysozoa or animals as a whole. We predict four types of start codons for protein-coding genes in *E*. *svetlanae* and five in *P*. *kielensis* with a consensus DTD in single letter code. The mitochondrial genomes of *E*. *svetlanae* and *P*. *kielensis* encode duplicated methionine tRNA genes that display compensatory nucleotide substitutions. Two distant species of Kinorhyncha demonstrate similar patterns of gene arrangements in their mitogenomes. Both genomes have duplicated methionine tRNA genes; the duplication predates the divergence of two species. The kinorhynchs share a few features pertaining to gene order that align them with Priapulida. Gene order analysis reveals that gene arrangement specific of Priapulida may be ancestral for Scalidophora, Ecdysozoa, and even Protostomia.

## Introduction

Mitochondrial genomes provide a set of important tools for evolutionary studies of animals owing to their accessibility and higher evolutionary rate in comparison with the nuclear genomes. The animal mitochondrial genomes are compact and typically encode 13 proteins of the respiratory chain (nad1-6, nad4L, cox1-3, cytb, atp6, and atp8), two subunits of ribosomal RNA and a variable complement of transfer RNAs on a circular DNA molecule with an average size of about 15 Kbp [1, 2]. In addition to the gene sequence data, the mitochondrial genome displays other features that are also applicable for evolutionary studies. Although the gene content of animal mitogenomes is nearly constant, the arrangement of these genes on the DNA molecule varies among major taxonomic groups. It was suggested that changes in the mitochondrial gene order can be used as cladistic characters for studying relationships among higher-level taxa [3]. In some taxonomic groups such as insects and vertebrates the gene order tends to be conservative, while in some other groups such as Mollusca [4], Bryozoa [5–7], Acari [8], and Tunicata [9, 10] it is highly variable. Generally, the taxon-specific gene order remains identical over long periods of time [11–13]. Furthermore, the analyses of gene order in bilaterian mitogenomes revealed conservation of specific gene blocks encompassing both protein-coding and ribosomal RNA genes [14]. The gene order within blocks remains uniform in most species, while the blocks themselves experience transpositions. The arrangements of these blocks allow to deduce putative ground patterns for some bilaterian taxa, including Ecdysozoa [15], Lophotrochozoa [16], and Deuterostomia [17]. However, the ground patterns for taxa with highly variable gene order (such as Nematoda or Chaetognatha) or for higher-level taxa (such as Bilateria or Metazoa) remains intractable. Within Ecdysozoa the mitochondrial gene order is generally more variable than in other taxa of the same rank, and it is potentially a valuable resource for phylogenetic information in the group [18–20].

The phylogenetic relationships within Ecdysozoa remain contradictory [21]. One of the important for evolutionary studies but relatively poorly studied ecdysozoan groups is Kinorhyncha. It is a small phylum of free-living, meiobenthic segmented pseudocoelomate worm-like invertebrates that accommodates 222 described species [22, 23] distributed worldwide. Usually they are much smaller than 1 mm, but a few Arctic species reach the size of 1.1 mm. They inhabit the upper layers of marine sediment with densities of 1–10 animals per 10 cm^2^ in the deep sea regions and 45 animals per 10 cm^2^ in the shallow regions [24, 25]. Taxonomically, Kinorhyncha is classified with Priapulida and Loricifera into a clade Scalidophora on the basis of morphological similarities [26]. The resent fossil findings point to early divergence of these three phyla—about 535 million years ago in the Cambrian Period [27]. Currently, the monophyly of a group uniting Kinorhyncha and Priapulida is confirmed by ribosomal phylogenies [28–30] and multigene phylogenetic analyses [31, 21]. However, only two mitochondrial genomes from the Scalidophora lineage have been sequenced to date, both from representatives of phylum Priapulida [15, 32].

**Fig 1:**
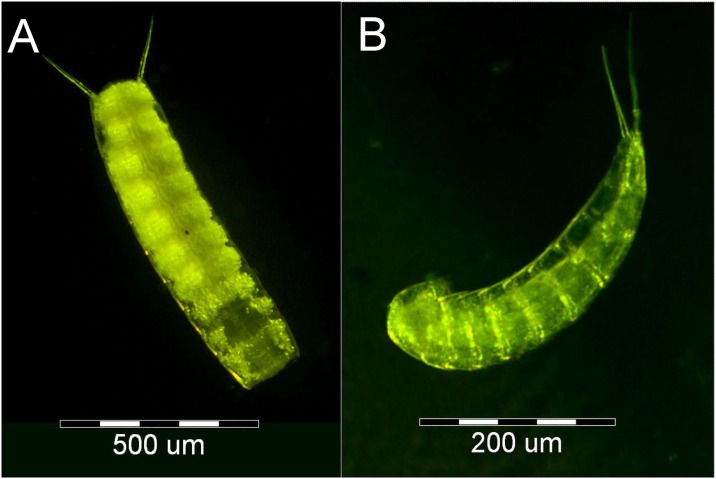
Eternal morphology of kinorhynchs. **A.** *Pycnophyes kielensis*, scale bar = 500 μm; **B.** *Echinoderes svetlanae*, scale bar = 200 μm. Heads orient down.

Here we present mitochondrial genomes of two kinorhynchs belonging to orders Cyclorhagida (*Echinoderes svetlanae*) and Allomalorhagida (*Pycnophyes kielensis*) (Fig 1).

## Materials and Methods

### Material collection and DNA extraction

Adult individuals of *Pycnophyes kielensis* were collected by bubbling from the upper layers of marine sediment in the vicinity of the Kartesh Biological Station, Zoological Institute, Russian Academy of Sciences (Chupinskaya Bight of Kandalaksha Bay, White Sea) and delivered live to the laboratory. Specimens of *Echinoderes svetlanae* were caught by trawling (Rugozerskaya Inlet, Kandalaksha Bay, White Sea) in the vicinity of the White Sea Biological Station of Moscow State University, collected by bubbling, and fixed by ethanol. Permissions to fieldwork were issued by the authority of Pertsov White Sea Biological Station of Lomonosov Moscow State University and Kartesh Biological Station of Zoological Institute of Russian Academy of Sciences. Field studies did not involve endangered or protected species.

Tissues of kinorhynchs were homogenized in 100 μl of buffer solution containing 0.01 M TrisHCl, 0.1 M EDTA, and 0.15 M NaCl (pH 8.0) and then lysed with 0.5% sodium dodecyl sulfate (SDS) and Pronase (100 μg/ml) at 37°C. DNA of both species was isolated from a few dozens of individuals using the DIAtom DNA Prep kit (Isogen, Russia) following the protocol provided by the manufacturer.

### Mitochondrial genome sequencing, assembly and annotation

Genomic DNA libraries for *P*. *kielensis* and *E*. *svetlanae* were prepared with Nextera DNA Sample Preparation Kit (Illumina, Illinois, USA) and sequenced on a HiSeq2000 instrument. The paired-end reads were adapter trimmed with Trimmomatic 0.30 [33] and assembled with SPAdes 3.5.0 [34]. The mitogenome sequences were detected in the assemblies by BLAST searches [35] and annotated using the MITOS web server [36]. Transfer RNA genes were detected using the MiTFi program [37], and their secondary structures were predicted using the MITOS web server. The annotations were refined manually using alignments of protein-coding sequences and the GenomeView browser [38]. Mitochondrial genome maps (S1 and S2 Figs) were constructed using OGDRAW software [39].

### Genetic code and nucleotide composition

AT and GC skew were determined for the complete mitochondrial genomes (major strand) according to the formula AT-skew = (A − T) / (A + T) and GC-skew = (G − C) / (G + C) [40], where the letters stand for the absolute number of the corresponding nucleotides in the sequences. Genetic code and codon usage were analyzed by GenDecoder v1.6 [41] and FACIL [42] web tools. Characterization of codon usage bias was calculated with the BioEdit program [43].

### Gene order analysis

We used CREx [44] for pairwise comparisons of kinorhynch mitogenomes between each other and with putative ground patterns for Panarthropoda, Priapulida, Lophotrochozoa and Deuterostomia constructed on the basis of conservative gene blocks described in [14]. CREx determines the most parsimonious genome rearrangement scenario given the gene order of two genomes, accounting for transpositions, reverse transpositions, reversals, and tandem-duplication random-loss events. The most parsimonious rearrangement scenarios for user trees were found by TreeREx [45]. The mitochondrial genomes from the OrganelleResource database of NCBI [46] were used for comparison with kinorhynch gene orders. The ground patterns for groups Panarthropoda, Lophotrochozoa, and Deuterostomia were obtained from previous studies [15–17]. Duplicated *trnM* genes in mitochondrial genomes were found using mitotRNAdb [47].

### Phylogenetic analysis

The protein-coding gene sequences of *E*. *svetlanae* and *P*. *kielensis* were translated using the invertebrate mitochondrial genetic code and aligned with sequences from 80 metazoans, selected from the NCBI’s organelles genome database (S1 Table). We excluded Nematoda from the phylogenetic analysis to decrease the influence of the long branch attraction (LBA) artefact [14, 32].

The aminoacid sequences were aligned with MAFFT v7.130 [48] using the pairwise Needleman-Wunsch algorithm (—globalpair) with the offset parameter set to 0.123 (—ep 0.123) and the maximum number of iterative refinement set to 1000 (—maxiterate 1000). The alignments were trimmed with trimAl 1.2rev57 [49] using a gap threshold of 0.9 and a similarity threshold of 0.0005 over a window of size 3 (-w 1), and concatenated using SCaFoS 1.25 [50]. Phylogenetic inference was performed by PhyloBayes MPI 1.5a [51] after removing constant positions and fast-evolving sites from the alignment. The evaluation of site-wise evolutionary rates was performed with TREE-PUZZLE 5.3rc16 [52] under the MtZoa model [53], and quarter of sites were removed from the alignment starting with the fastest evolving category. The PhyloBayes tree inference was performed with 4 independent Monte Carlo Markov chains running for 20,000 cycles under the MtZoa model with a site-specific profile mixture (CAT) [54] and across-site rate variation modeled by discrete Gamma distribution with 4 categories. The majority rule consensus tree was constructed from the trees sampled every 10 cycles after discarding 50% of them as burn-in. The alternative topologies were tested using the approximately unbiased (AU) test implemented by the CONSEL program [55]. The alternative topologies were constructed using MEGA 5.2.2 [56], and corresponding site-wise log likelihood values for them were computed with TREE-PUZZLE under the MtZoa model.

The nucleotide sequence alignments were constructed on the basis of the aminoacid alignments using the TranslatorX program [57], trimmed according to the mask derived from the trimmed aminoacid alignments and concatenated with SCaFoS. The inferences with the nonstationary models BP and BP+CAT were performed with NH PhyloBayes 0.2.3 [58], and the inferences with the CAT and CAT+GTR models were performed with PhyloBayes 4.1c [54]. Each analysis was run in 2 independent chains, the analyses with the CAT and CAT+GTR models were sampled across 20,000 cycles and the analyses with the nonstationary models were sampled across 200 points; the trees were summarized with a 10% burn-in.

Transfer RNA gene duplication was analyzed by phylogenetic approach. Each tRNA was divided into acceptor, anticodon, D- and T-arm regions according to the predicted secondary structure obtained by MiTFi [37]. Each stem or loop region was aligned with the corresponding region from other tRNAs and alignments were concatenated. Concatenated tRNA alignment was minimized to a mask of 52 bp. A NEXUS file was generated from the alignment by the STEMS program [59] accounting the predicted secondary structure and creating stem and loop partitions. Phylogenetic inference for tRNA genes was performed by MrBayes v.3.2.5 [60]. The evaluation of site-wise evolutionary rates was performed under the four-by-four model with covarion for the loops partition and the doublet model without covarion for the stems partition. Two independent runs of four Markov Chain Monte Carlo (MCMC) were performed for 1,000,000 generations, sampling trees every 1,000 generations. The first 500 trees were discarded as burn-in, and the remaining set was used to generate a consensus tree with posterior probability values. The resulting Bayesian tree was visualized in MEGA 5.2.2 [56].

## Results and Discussion

### Mitochondrial genome organization and nucleotide composition

Mitochondrial genomes of *Echinoderes svetlanae* (GenBank KU975552) and *Pycnophyes kielensis* (GenBank KU975551) are closed circular DNA molecules with lengths of 15304 bp and 14985 bp respectively. The kinorhynch mitogenomes have a rather low GC content of about 26% in both species (Table 1), although the lowest known GC content in mitochondrial genomes is 12,6% [14]. The GC content in tRNA and rRNA genes of kinorhynchs is even lower relative to the whole mitochondrial genome (Table 2), but in protein-coding genes (PCGs) the GC content slightly exceeds the average. The non-coding regions in both species have a lower GC content than the whole genome average. The GC and AT skews characterize the asymmetry of nucleotide content between the two strands of mitochondrial DNA [40, 61]. The prevalence of thymine over adenine and guanine over cytosine in the major strands provides negative AT-skew and positive GC-skew in kinorhynchs similarly to most other animals [14].

**Table 1:** Nucleotide composition characteristics of *E*. *svetlanae* and *P*. *kielensis* mitochondrial genomes.

Species	GC%	A%	T%	G%	C%	AT-skew	GC-skew
*E*. *svetlanae*	26	28	47	18	8	-0,26	0,39
*P*. *kielensis*	26	32	42	19	7	-0,14	0,44

**Table 2:** GC% contents of *E*. *svetlanae* and *P*. *kielensis* mitochondrial genomes.

Species	Whole genome	PCG	tRNA	rRNA	Total non-coding region
*E*.*svetlanae*	26	27	21	22	19
*P*.*kielensis*	26	28	20	21	21

The mitogenomes of *E*. *svetlanae* and *P*. *kielensis* contain a common set of 37 metazoan mitochondrial genes (13 PCGs, 22 tRNA genes, and two rRNA genes) and one additional methionine tRNA gene (Tables 3 and 4). All genes in both species are located in the major strand.

**Table 3:** *Pycnophyes kielensis* genome organization.

Gene	Strain	Position (start-stop)	Length (bp)	Intergenic space (bp)	Start codon	Stop codon
*cox1*	+	3–1586	1584	19	GTG	TAA
*trnL1*	+	1606–1667	62	3		
*trnA*	+	1671–1732	62	-5		
*trnS1*	+	1728–1789	62	3		
*trnF*	+	1793–1848	56	1		
*trnS2*	+	1850–1915	66	3		
*trnL2*	+	1919–1980	62	6		
*trnE*	+	1987–2049	63	238		
*trnV*	+	2288–2350	63	15		
*rrnS*	+	2366–3098	733	0		
*trnG*	+	3099–3160	62	54		
*atp8*	+	3215–3358	144	2	ATG	TAA
*trnK*	+	3361–3421	61	18		
*rrnL*	+	3440–4421	982	104		
*nad3*	+	4526–4852	327	4	ATA	TAA
*cytb*	+	4857–5999	1143	18	GTG	TAA
*nad4*	+	6018–7313	1296	9	ATA	TAA
*nad1*	+	7323–8207	885	245	ATT	TAG
*nad2*	+	8453–9442	990	22	ATA	TAA
*trnQ*	+	9465–9529	65	0		
*trnM1*	+	9530–9592	63	4		
*trnC*	+	9597–9656	60	0		
*trnI*	+	9657–9718	62	4		
*trnP*	+	9723–9784	62	9		
*nad4l*	+	9794–10069	276	-11	ATA	TAA
*trnD*	+	10059–10120	62	3		
*trnT*	+	10124–10185	62	14		
*cox2*	+	10200–10889	690	56	TTG	TAG
*atp6*	+	10946–11590	645	10	ATG	TAA
*trnN*	+	11601–11663	63	35		
*cox3*	+	11699–12478	780	9	ATA	TAA
*trnM2*	+	12488–12553	66	0		
*nad6*	+	12554–13018	465	4	ATA	TAA
*trnY*	+	13023–13082	60	4		
*trnR*	+	13087–13149	63	3		
*trnH*	+	13153–13213	61	11		
*trnW*	+	13225–13287	63	6		
*nad5*	+	13294–14982	1689	5	ATA	TAA

**Table 4:** *Echinoderes svetlanae* genome organization.

Gene	Strain	Position (start-stop)	Length (bp)	Intergenic space (bp)	Start codon	Stop codon
*cox1*	+	6–1577	1572	270	ATG	TAA
*trnE*	+	1848–1912	65	0		
*trnL2*	+	1913–1982	70	4		
*rrnS*	+	1987–2752	766	11		
*trnG*	+	2764–2833	70	81		
*trnV*	+	2915–2981	67	4		
*trnL1*	+	2986–3059	74	-18		
*atp8*	+	3042–3221	180	7	ATA	TAG
*trnK*	+	3229–3295	67	142		
*rrnL*	+	3438–4451	1014	34		
*nad1*	+	4486–5397	912	4	ATT	TAA
*nad2*	+	5402–6430	1029	4	ATG	TAA
*trnQ*	+	6435–6502	68	-4		
*trnM1*	+	6499–6566	68	5		
*trnI*	+	6572–6640	69	5		
*trnC*	+	6646–6707	62	9		
*trnP*	+	6717–6786	70	18		
*nad4l*	+	6805–7077	273	5	ATA	TAG
*trnD*	+	7083–7146	64	2		
*trnA*	+	7149–7214	66	6		
*trnT*	+	7221–7288	68	0		
*cox2*	+	7289–7966	678	10	TTG	TAA
*trnN*	+	7977–8046	70	12		
*atp6*	+	8059–8745	687	17	ATA	TAA
*cox3*	+	8763–9572	810	27	ATG	TAA
*trnS1*	+	9600–9661	62	4		
*trnM2*	+	9666–9728	63	18		
*nad6*	+	9747–10226	480	55	ATA	TAA
*nad3*	+	10282–10608	327	11	ATA	TAA
*cytb*	+	10620–11774	1155	9	ATT	TAA
*nad4*	+	11784–13124	1341	11	ATG	TAG
*trnS2*	+	13136–13205	70	6		
*trnY*	+	13212–13279	68	5		
*trnR*	+	13285–13351	67	0		
*trnF*	+	13352–13421	70	4		
*trnH*	+	13426–13494	69	1		
*trnW*	+	13496–13564	69	51		
*nad5*	+	13616–15301	1686	8	ATG	TAA

The non-coding regions in the mitogenome of *E*. *svetlanae* are 860 bp in total and consist of 33 intergenic segments, ranging from 1 to 270 bp and include two major non-coding regions of more than 100 bp. The non-coding part of *P*. *kielensis* mitogenome consists of 32 intergenic segments ranging from 1 to 245 bp and totaling 941 bp, where three major non-coding regions have a length of more than 100 bp.

The mitogenome of *P*. *kielensis* has two gene overlaps: the first one is between *nad4l* and *trnD* (11 bp) and the second one is between *trnA* and *trnS1* (5 bp). In the mitogenome of *E*. *svetlanae* there are also two overlaps: one between *trnL1* and *atp8* (18 bp) and another between *trnQ* and *trnM1* (4bp).

Excluding all termination codons, the cumulative length of 13 PCGs of *E*. *svetlanae* is 10446 bp, encoding 3722 amino acid residues. The cumulative length of PCGs of *P*. *kielensis* is 10650 bp, encoding 3637 amino acid residues. The GC% at the first two codon positions exceeds the average GC% for the whole mitochondrial genome (Tables 2 and 5), and the GC% at the third codon position is very low– 14% and 16% for *E*. *svetlanae* and *P*. *kielensis* respectively.

**Table 5:** GC% in the three codon positions of *E*. *svetlanae* and *P*. *kielensis* PCGs.

Codon position	GC%	
	*E*. *svetlanae*	*P*. *kielensis*
1	35	36
2	33	33
3	14	16

Transfer RNA genes in kinorhynchs are distributed throughout the circular molecule and have a total length of 1556 bp in *E*. *svetlanae* and 1431 bp in *P*. *kielensis*. Mitochondrial tRNAs range from 62 bp to 74 bp in *E*. *svetlanae* and from 56 bp to 66 bp in *P*. *kielensis* (Tables 3 and 4). The secondary structures of the *E*. *svetlanae* and *P*. *kielensis* tRNAs predicted by MiTFi are shown in additional files (S3 and S4 Figs). All tRNA genes were folded into the typical cloverleaf secondary structure, except for the *E*. *svetlanae trnS1* gene, where the dihydrouridine arm is simplified to a loop, and the *P*. *kielensis trnF* with a loop instead of the TΨC arm (the T-loop).

Two rRNA genes (*rrnL* and *rrnS*) are located on the major strands. The *rrnL* gene is located after the *trnK* in both species and before *nad1* gene in *E*. *svetlanae* or *nad3* gene in *P*. *kielensis*. The *rrnS* gene is located before *trnG* gene in both species and after *trnL2* gene in *E*. *svetlanae* or *trnV* gene in *P*. *kielensis* (Tables 3 and 4). The length of the *rrnL* gene is 1014 bp in *E*. *svetlanae* and 982 bp in *P*. *kielensis*. The length of the *rrnS* gene is 766 bp in *E*. *svetlanae* and 733 bp in *P*. *kielensis*.

### Duplicated tRNA genes

The overwhelming majority of metazoan mitogenomes include two tRNA genes for serine and leucine and only one tRNA gene for each of the other 18 amino acids [62, 63]. Two serine and leucine tRNA genes are associated with two codon groups coding each of these amino acids: each tRNA anticodon is adapted for recognition of a codon group.

**Fig 2:**
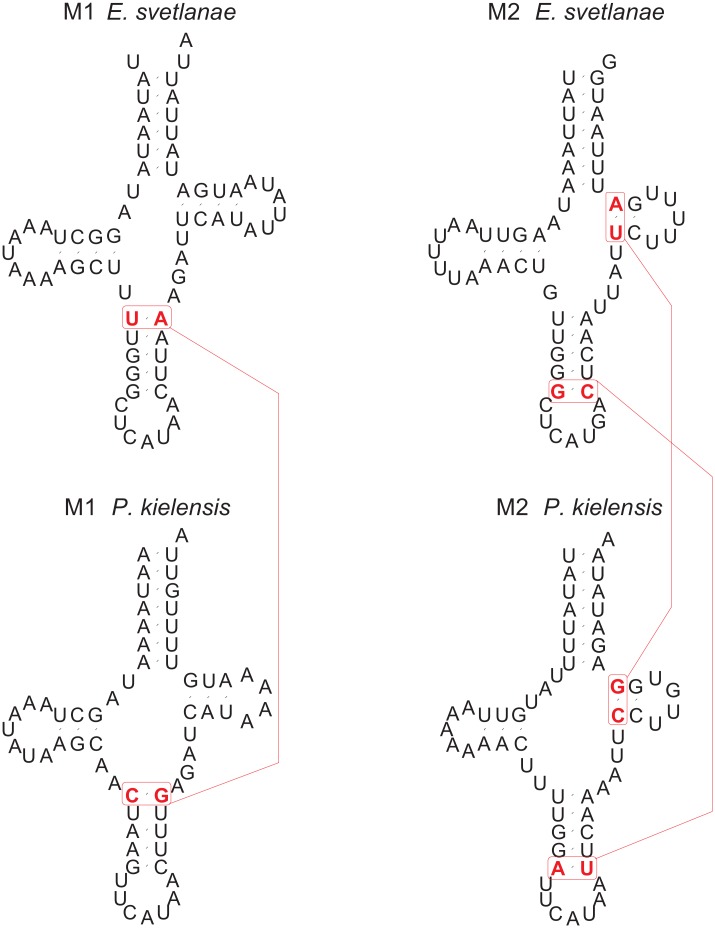
Predicted mitochondrial methionine tRNAs of *Echinoderes svetlanae* and *Pycnophyes kielensis*. Compensatory changes are shown in red. Compensatory change in M1 –the first pair of the anticodon stem (U-A in *E*. *svetlanae* and C-G in *P*. *kielensis*). Compensatory changes in M2 –the first pair of the T-arm (U-A in *E*. *svetlanae* and C-G in *P*. *kielensis*) and the fifth pair of the anticodon stem (G-C in *E*. *svetlanae* and A-U in *P*. *kielensis*).

The mitogenomes of both *E*. *svetlanae* and *P*. *kielensis* encode two methionine tRNA genes (Fig 2). The tRNA M1 gene in both species is located after a glutamine tRNA gene, and the M2 gene—before *nad6* gene (Tables 3 and 4). Priapulids *Priapulus caudatus* [15] and *Halicryptus spinulosus*, which are presumed to be the closest relatives of kinorhynchs, have only one methionine tRNA gene which is positioned after a glutamine tRNA gene like the *trnM1* of *E*. *svetlanae* and *P*. *kielensis*.

The predicted kinorhynch methionine tRNAs have a classic “clover leaf” secondary structure with two lateral arms, and their loops and stems have similar sizes. There are two compensatory changes in the M2 tRNA helices of *E*. *svetlanae* and *P*. *kielensis*, and in M1 tRNAs there is one compensatory change (Fig 2). The presence of these compensatory changes strongly suggests that kinorhynch methionine tRNAs are not pseudogenes.

**Fig 3:**
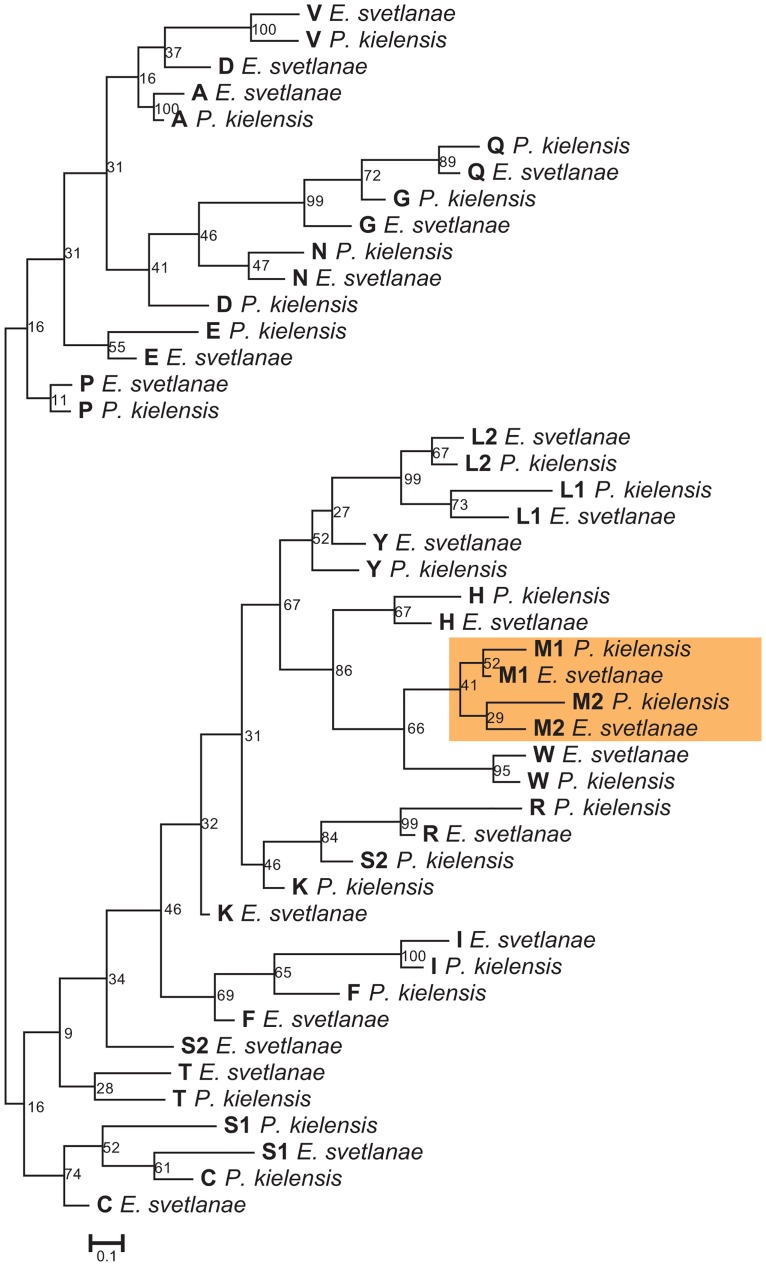
Bayesian tree based on the alignment of tRNA genes from *E*. *svetlanae* and *P*. *kielensis*. Numbers at the branches indicate Bayesian posterior probabilities. Methionine tRNA genes are marked orange. tRNA specificity is coded by one letter.

The Bayesian inference with tRNA genes of *E*. *svetlanae* and *P*. *kielensis* revealed an association between the same amino acids from both species for the majority of tRNAs including two pairs of methionine tRNA genes (Fig 3). The observed placement of *trnM1* and *trnM2* suggests that the additional gene originated by duplication of the methionine tRNA gene before the divergence of *E*. *svetlanae* and *P*. *kielensis*. The ancient nature of this duplication is also evidenced by the large difference in the primary structures of paralogous genes and the T-arm length difference in the predicted secondary structures. The functional significance of mitochondrial tRNA-Met gene duplication in kinorhynchs is not clear, but it might represent different functions of the methionine tRNAs in protein synthesis and initiation of translation. Note that in plastid genomes the presence of two tRNA-Met genes is relatively common [64]: one methionine tRNA functions during the elongation phase of protein synthesis, and the other is charged with formyl methionine to function as an initiator tRNA. In mitochondrial genomes the duplication of *trnM* was also found in some placozoans, cnidarians, insects, vertebrates. In molluscs and tunicats, each methionine tRNA, tRNA-Met(AUG) and tRNA-Met(AUA), can recognize a specific methionine codon instead of one tRNA with AUR specificity [65–67]. In some sponges the product of one *trnM* gene is post-transcriptionally edited to function as an additional isoleucine tRNA [68, 69].

Besides the *trnM* there are other rare cases of mitochondrial tRNA duplications. For example, a duplicated *trnL* (CUN) was found in the Japanese freshwater crab *Geothelphusa dehaani* [70] and a duplicated *trnI* is found in three blow-fly species from genus *Chrysomya* [71]. In other cases one copy of the duplicated tRNA gene accumulates multiple mutations and undergoes deletions decreasing the gene length in comparison with functional tRNAs [70, 71]. Thus, one of the duplicated isofunctional tRNA genes may become a pseudogene that is eventually eroded from the mitogenome. Duplicated tRNAs may also appear as a result of tRNA remolding (the change of tRNA specificity)–like in gecko *Tropiocolotes tripolitanus* with two *trnQ*, one of which has evolved from *trnR* [72]. However in this case the duplicated genes strongly differ in primary and secondary structures and the remolded tRNA likely remains as an inactive form.

### Genetic code and codon usage

The kinorhynch mitochondrial PCGs do not contain all of the possible codons (Table 6). There are no CTC and CTG codons for leucine and TCC codon for serine in *E*. *svetlanae*. The PCGs of *P*. *kielensis* have no arginine codon CGC.

**Table 6:** The codon usage in *E*. *svetlanae* and *P*. *kielensis*.

Amino Acid	Codon	*E*. *svetlanae*	*P*. *kielensis*	Amino Acid	Codon	*E*. *svetlanae*	*P*. *kielensis*
A	GCA	28	45	F	TTC	5	18
	GCC	5	8		TTT	425	395
	GCG	6	13	L	TTA	389	365
	GCT	99	53		TTG	66	64
R	CGA	23	41		CTA	14	28
	CGC	1	-		CTC	-	3
	CGG	7	15		CTG	-	5
	CGT	53	9		CTT	38	37
Y	TAT	204	151	I	ATC	4	12
	TAC	14	10		ATT	286	254
N	AAT	115	82	V	GTA	71	155
	AAC	9	4		GTC	4	6
D	GAT	90	74		GTG	39	62
	GAC	5	7		GTT	186	116
C	TGT	66	34	S	TCA	30	46
	TGC	2	3		TCC	-	4
E	GAA	43	64		TCG	4	9
	GAG	39	38		TCT	111	64
P	CCA	23	22		AGT	92	22
	CCC	1	6		AGC	6	4
	CCG	9	7		AGA	50	143
	CCT	75	47		AGG	43	41
T	ACA	32	35	H	CAT	68	47
	ACC	3	6		CAC	5	10
	ACG	4	3	K	AAA	41	59
	ACT	62	55		AAG	28	20
Q	CAA	33	27	M	ATG	55	57
	CAG	13	13		ATA	185	283
G	GGA	42	137	W	TGA	63	91
	GGC	20	3		TGG	35	18
	GGG	90	122	Stop codons	TAA	10	11
	GGT	163	65		TAG	3	2

Both GenDecoder and FACIL programs determined that the majority of kinorhynch codons code the same amino acids as the standard invertebrate mitochondrial code. However, they also found some differences from this code in both species (S2 Table). GenDecoder specified the TGC codon as proline in *E*. *svetlanae* and as alanine in *P*. *kielensis*. The ACG codon is specified by GenDecoder as serine in *P*. *kielensis*, and as threonine in *E*. *svetlanae*. In *E*. *svetlanae* FACIL specified TTC as leucine and TGT as alanine, and in *P*. *kielensis* it specified the ATC codon as leucine.

**Fig 4:**
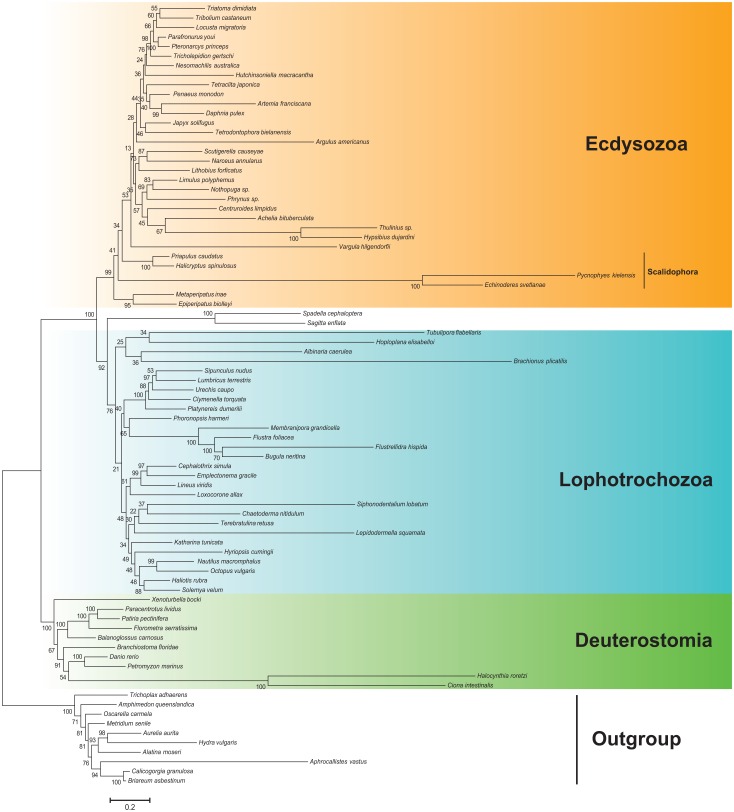
Bayesian tree based on the concatenated dataset of 13 protein-coding genes from mitochondrial genomes after removing constant positions and fast-evolving sites from the alignment. Numbers at the branches indicate Bayesian posterior probabilities as percent values.

Likewise, there are some differences in specifying the ATG and ATA codons that normally code methionine in invertebrate animals. FACIL specified these codons in both species as leucine, and GenDecoder specified the ATG as leucine in *E*. *svetlanae*. Considering that kinorhynchs have two tRNA genes with a conventional methionine anticodon it is possible that one of these tRNAs may be actually charged with leucine. In addition, the long branches of kinorhynchs in the phylogenetic tree (Fig 4) may be partially due to incorrect translation of ATG codons. It is possible that kinorhynch *trnM*s are charged by different amino acids (methionine or leucine), and introduce them to the protein chain randomly or deterministically. However our data do not allow us to draw a clear conclusion about the exact specification of these codons.

The results of codon predictions by the both programs do not completely coincide in two kinorhynch species. Four of the seven controversial codons (TGC, ACG, TTC and ATC) analyzed by the programs are very rare in both species and consequently may be specified mistakenly. We compared three other controversial codons with tRNA anticodons found in the kinorhynch mitochondrial genomes. The TGT codon does not form a complementary pair with the alanine tRNA at all three codon positions. The ATG and ATA codons can form two of the three complementary bonds with the leucine tRNAs. At this point it is not possible to answer whether we are observing a different genetic code in kinorhynch mitogenomes or whether the deviations detected by the genetic code analysis have other explanations and we are actually dealing with a standard invertebrate mitochondrial code. Previous research on *cox1* predicted the invertebrate mitochondrial code in kinorhynchs [73].

The codon usage in the mitochondrial genomes of *E*. *svetlanae* and *P*. *kielensis* shows a strong preference for synonymous codons ending with thymine or adenine (Table 6). *E*. *svetlanae* and *P*. *kielensis* favor the NNT codon from the NNN or NNY codon families and the NNA from the NNR codon families. All of the missing codons end with guanine or cytosine and consist of guanine or cytosine completely or by 2/3.

We predict five types of start codons in kinorhynch species (Table 7). The majority of PCGs are predicted to start with codons ATG or ATA. However, based on the amino acid alignments it is clear that additional codons are likely employed for translation initiation in kinorhynchs. Additional start codons are specific for some genes: *cox2* is likely initiated by the leucine codon TTG whereas *nad1* is likely initiated by the isoleucine codon ATT in both species; *cytb* is likely initiated by different additional codons in both species (isoleucine codon ATT in *E*. *svetlanae* and valine codon GTG in *P*. *kielensis*). Moreover, *cox1* in *P*. *kielensis* is likely initiated by a valine codon GTG. All these codons have been already mentioned to function as start codons in some mitochondrial PCGs, for example, TTG in *Epinephelus coioides* [74], GTG in *Tylototriton taliangensis* [75], *Scapanulus oweni* [76] and *Euthynnus affinis* [77], ATT in *S*. *oweni*. Additional start codons are not rare in mitochondrial genomes, but the presence of four (in *E*. *svetlanae*) or five (in *P*. *kielensis*) start codons is unusual. There are two stop codons (TAA and TAG) in kinorhynch species (Table 7). Both species prefer the stop codon TAA ending with an adenine.

**Table 7:** Start and stop codon occurrence in PCG of *E*. *svetlanae* and *P*. *kielensis*.

	Start codon					Stop codon	
	ATA	ATG	TTG	GTG	ATT	TAA	TAG
*E*. *svetlanae*	5	5	1	-	2	10	3
*P*. *kielensis*	7	2	1	2	1	11	2

The positive GC skew and negative AT skew of the coding strand affects the amino acid composition bias in PCGs. In kinorhynchs there is an excess amount of amino acids coded by GT-rich codons: Phe, Gly, Val and Trp. At the same time the number of amino acids coded by AC-rich codons (Thr, Pro, Asn, His and Gln) is noticeably lower.

### Phylogenetic analysis

The Bayesian inference with concatenated mitochondrial protein alignments recovers well-supported conventional monophyletic groups of Deuterostomia, Lophotrochozoa, and Ecdysozoa (S5 Fig). Two kinorhynch species are grouped together forming a long branch, which is placed within arthropods contradicting the modern conceptions of ecdysozoan taxa. To decrease the possible impact of long-branch attraction (LBA) caused by divergent kinorhynch sequences, we applied the removal of fast-evolving sites from the alignment. After removal of the fastest evolving sites from the protein alignment, the conventional taxa have retained their monophyly and the long branch of kinorhynchs was positioned near the root of Ecdysozoa (Fig 4). However, we did not observe the kinorhynchs group with Priapulida (*Priapulus caudatus* and *Halicryptus spinulosus*) in what would constitute the monophyletic Scalidophora, contrary to the results obtained with rRNA or nuclear protein coding gene datasets [28–31, 21]. The LBA artifacts in mitochondrial PCGs were noted in other ecdysozoans [32], and, perhaps, the observed placement of kinorhynchs in the mitochondrial protein tree is associated with their accelerated evolutionary rate. It was observed that the basal branching in Ecdysozoa is highly sensitive to taxon sampling and the choice of model for the mitogenome data [32]. In our analysis the first branch of Ecdysozoa is Onychophora with posterior probability 0.41. Generally the basal position of onychophores is not supported by molecular phylogeny, but an onychophore-like ancestor was suggested for Ecdysozoa by paleontological data [78].

We tested whether the alternative positions of Kinorhyncha in the mitochondrial PCGs tree are significantly worse than the reconstructed topology. The majority of tested alternative topologies differ insignificantly from the Bayesian tree. The tree with the monophyletic Scalidophora clade has the AU-test *p*-value of 0.563, and the tree with Scalidophora as the basal branch within Ecdysozoa has the AU-test *p*-value of 0.378. The results do not contradict the hypothesis of Scalidophora monophyly or the basal position of Scalidophora within Ecdysozoa. The low values of posterior probabilities and results of the AU-test indicate poor resolution of the mitochondrial ecdysozoan tree, which agrees with previous observations [32].

Because the poor resolution of the mitochondrial tree may be associated with the GC bias, we analyzed the nucleotide sequences that underlie the protein alignment using nonstationary models implemented by NH PhyloBayes as well as stationary PhyloBayes models. The Bayesian inference with nucleotide data revealed some differences with the aminoacid data inference depending on the employed model (S6–S9 Figs). The trees built with stationary models CAT or CAT+GTR (S6 and S7 Figs) are broadly similar and recover monophyletic Ecdysozoa, while Deuterostomia and Lophotrochozoa are split into several polyphyletic groups. Kinorhynchs are grouped with onychophores into a clade sister to priapulids (S6 Fig) or within arthropods (S7 Fig) with low posterior probability. Trees under the nonstationary models BP and CAT+BP are more similar to the aminoacid tree (S8 and S9 Figs): in the first tree Ecdysozoa and Lophotrochozoa monophyly are destroyed by the kinirhynchs position only, while in the second one all the major taxa are monophyletic. We conclude that the inference with our mitochondrial nucleotide data is facilitated by using the nonstationary models of sequence evolution, presumably by alleviating the negative effect of the compositional bias.

### Mitochondrial gene order and rearrangements

**Fig 5:**
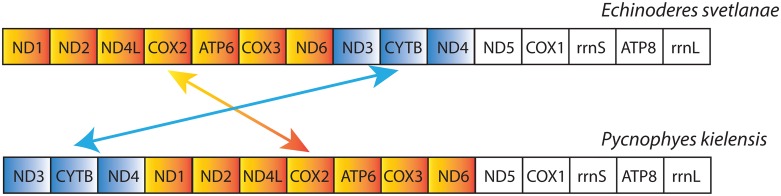
Protein-coding and rRNA gene orders in the mitochondrial genomes of *E*. *svetlanae* and *P*. *kielensis*.

The gene orders of *E*. *svetlanae* and *P*. *kielensis* mitochondrial PCGs are shown in Fig 5. These two arrangements differ by one transposition between two gene groups: *nad1-nad2-nad4l-cox2-atp6-cox3-nad6* and *nad3-cytb-nad5*. Both kinorhynch gene orders are unique: there are no similar arrangements in the Organelle Resource database not only among the Ecdysozoa (1007 species in the database at the time of comparison), but also among Bilateria (5165 species).

**Fig 6:**
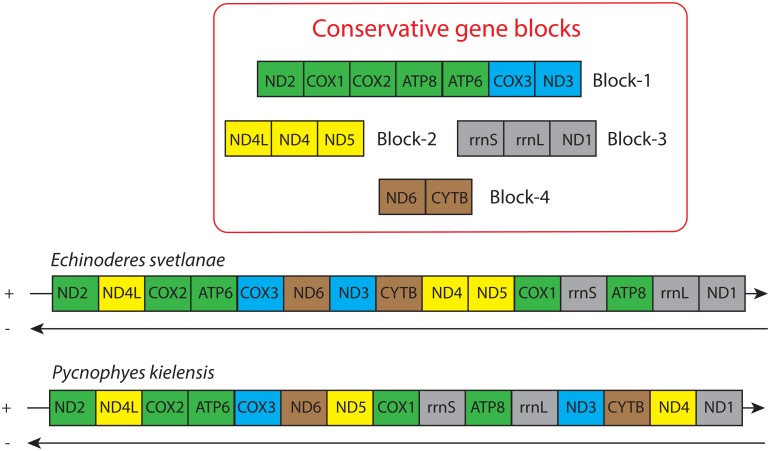
Kinorhynch gene orders and conservative blocks of mitochondrial genes from Bilateria. Genes and blocks are colored and named following [14].

**Fig 7:**
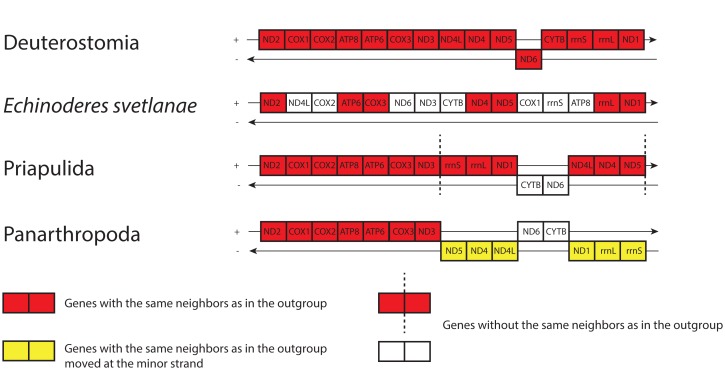
Gene orders of *E*. *svetlanae*, Priapulida and Panarthropoda [15] with Deuterostomia [17] as an outgroup.

Unlike most bilaterians, *E*. *svetlanae* and *P*. *kielensis* have gene orders that lack any of the previously described conservative gene blocks [14] (Fig 6). However, *E*. *svetlanae* has three pairs of adjacent genes from one conservative gene block–*cox2-atp6*, *nad4-nad5*, and *rrnL-nad1*, while *P*. *kielensis* has only one such pair of genes–*cox2-atp6*. This suggests that *E*. *svetlanae* has a more plesiomorphic gene order than *P*. *kielensis*. While all of the PCG blocks have been eroded in kinorhynchs, the conservative pairs *atp6-cox3* and *nad1-nad2*, which represent block boundaries, are preserved as common features in kinorhynchs and a majority of taxa within protostomes and deuterostomes (Fig 7). PCG location in the same chain is another widespread, although not universal character. We can assume that some of these features are inherited by kinorhynchs from a common ancestor of Bilateria.

We compared the gene orders and putative ground patterns of genes between several ecdysozoan groups and Deuterostomia as an outgroup (Fig 7). The most conservative gene clusters (*cox2-atp6*, *nad4-nad5*, and *rrnL-nad1-nad2*) are present in ecdysozoans and deuterostomes. As shown in Fig 7, Priapulida has the highest similarity to deuterostomes in their gene order. Some gene clusters in Panarthropoda are positioned in the minor strand, and *E*. *svetlanae* has the *rrnL-nad1-nad2* cluster, which is shared with deuterostomes but is exclusive among ecdysozoans.

**Fig 8:**
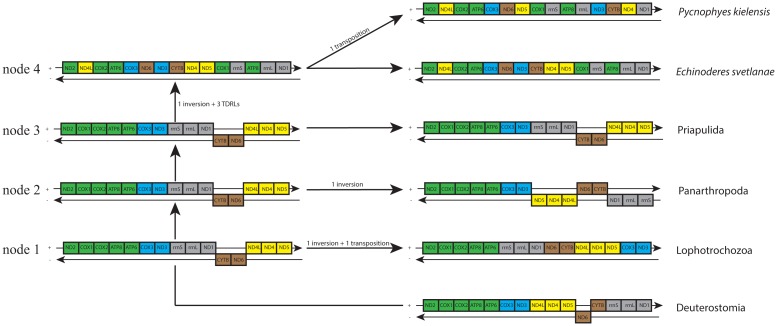
Putative model of gene order evolution in Protostomia reconstructed by TreeREx. Genes are colored following [14].

We reconstructed the gene order evolution in protostomian mitochondrial genomes using the TreeREx program and the previously suggested ground patterns of Panarthropoda [15], Lophotrochozoa [16], and Deuterostomia [17] (Fig 8). Deuterostomia was selected as an outgroup because their mitogenomes generally have more conservative gene orders than the mitogenomes of lophotrochozoans. The evolutionary scenario reconstructed by the TreeREx suggests that the ancestors of Scalidophora, Ecdysozoa, and Protostomia all share the same gene order, which coincides with the gene order seen in Priapulida, and proposes this pattern as a plesiomorphic trait in the group. Considering that the priapulid gene order is reconstructed as ancestral for both Scalidophora and Ecdysozoa, the kinorhynch gene order rearrangements are equally parsimonious under the monophyly of Kinorhyncha and Priapulida or under any alternative positions of Kinorhyncha around the base of Ecdysozoa, despite the fact the gene order of Panarthropoda can be converted to *E*. *svetlanae* in five steps while the gene order of Priapulida can be converted to *E*. *svetlanae* in four.

In the ancestors of some major clades, the genome rearrangements reshuffled the mitochondrial genes of the bilaterian ancestor generating patterns that have been described in terms of conservative gene blocks [14], which presumably correspond to fragments of the mitochondrial genome of the bilaterian ancestor. In other clades, such as kinorhynchs, the rearrangements reshuffle the ancestral genome to a point where the “block boundaries” seem to be more conservative than the “blocks”. The presence of taxa with highly altered gene orders in comparison to the presumed ancestral pattern, such as Nematoda [79], Chaetognata [80, 81], Acoela [82], and Urochordata [67, 83, 84] demonstrates the lack of strong selective restrictions on the patterns of the gene order. At the same time, the conservation of mitochondrial gene order patterns in various taxa indicates that the rearrangements are relatively rare events: the rate of rearrangements appears to be comparable to or lower than the rate of formation of the highest level taxa such as classes and phyla. Presumably, the gene order rearrangements are frequently deleterious rather than neutral. The gene arrangements differ greatly in the taxa with long branches in both mitochondrial and nuclear PCGs trees, such as nematodes, chaetognats, tunicates, and kinorhynchs. One of the possible reasons for the high rate fixation of unusual mutations simultaneously in coding sequences and gene pattern is the weakening of the purifying selection caused by the low effective population size [85–88] during the ancestor's history. Meiobenthic kinorhynchs have high populations and wide geographic distributions, but their patchy habitat may decrease the effective population size. Further studies are required to elucidate the reasons for the increased rate of evolution of mitochondrial sequences and gene orders in kinorhynchs and other taxa with divergent mitochondrial genomes.

## Conclusions

The complete mitochondrial genomes of two distant species of Kinorhyncha, *Echinoderes svetlanae* (Cyclorhagida) and *Pycnophyes kielensis* (Allomalorhagida), demonstrate similarity in the nucleotide composition, patterns of gene arrangements, and genome architecture. Both mitogenomes have duplicated methionine tRNA genes. The closest relatives of Kinorhyncha within Ecdysozoa are not clearly established by the mitochondrial PCGs phylogeny due to their highly divergent sequences, however the reconstructed scenario of gene order evolution does not contradict to the monophyly of Scalidophora. According to gene order analysis, Priapulida gene arrangement may be ancestral for Scalidophora, Ecdysozoa, and Protostomia.

## Supporting Information

S1 Fig Circular map of mitochondrial genome for *Echinoderes svetlanae*, GenBank KU975552. (PDF)

S2 Fig Circular map of mitochondrial genome for *Pycnophyes kielensis*, GenBank KU975551. (PDF)

S3 Fig Predicted mitochondrial tRNAs secondary structures of *Echinoderes svetlanae*. (PDF)

S4 Fig Predicted mitochondrial tRNAs secondary structures of *Pycnophyes kielensis*. (PDF)

S5 Fig Bayesian tree based on the concatenated dataset of 13 protein-coding genes from mitochondrial genomes without removing constant positions and fast-evolving sites from the alignment. Numbers at the branches indicate Bayesian posterior probabilities as percent.
(PDF)

S6 Fig Bayesian tree based on the concatenated nucleotide sequences of 13 PCGs under CAT model. Numbers at the branches indicate Bayesian posterior probabilities as percent.
(PDF)

S7 Fig Bayesian tree based on the concatenated nucleotide sequences of 13 PCGs under CAT+GTR model. Numbers at the branches indicate Bayesian posterior probabilities as percent values.
(PDF)

S8 Fig Bayesian tree based on the concatenated nucleotide sequences of 13 PCGs under BP model. Numbers at the branches indicate Bayesian posterior probabilities as percent values.
(PDF)

S9 Fig Bayesian tree based on the concatenated nucleotide sequences of 13 PCGs under CAT+BP model. Numbers at the branches indicate Bayesian posterior probabilities as percent values.
(PDF)

S1 Table Accession numbers of species downloaded from GenBank. (DOC)

S2 Table Differences in kinorhynchs mitogenomes from the invertebrate mitochondrial code predicted by GenDecoder and FACIL. (DOC)
