# Supplementary figures and images for: Mitochondrial Genomes of Kinorhyncha: trnM Duplication and New Gene Orders within Animals
Source: PLoS One. 2016 Oct 18;11(10):e0165072. doi: 10.1371/journal.pone.0165072 (PMC5068742; doi:10.1371/journal.pone.0165072)

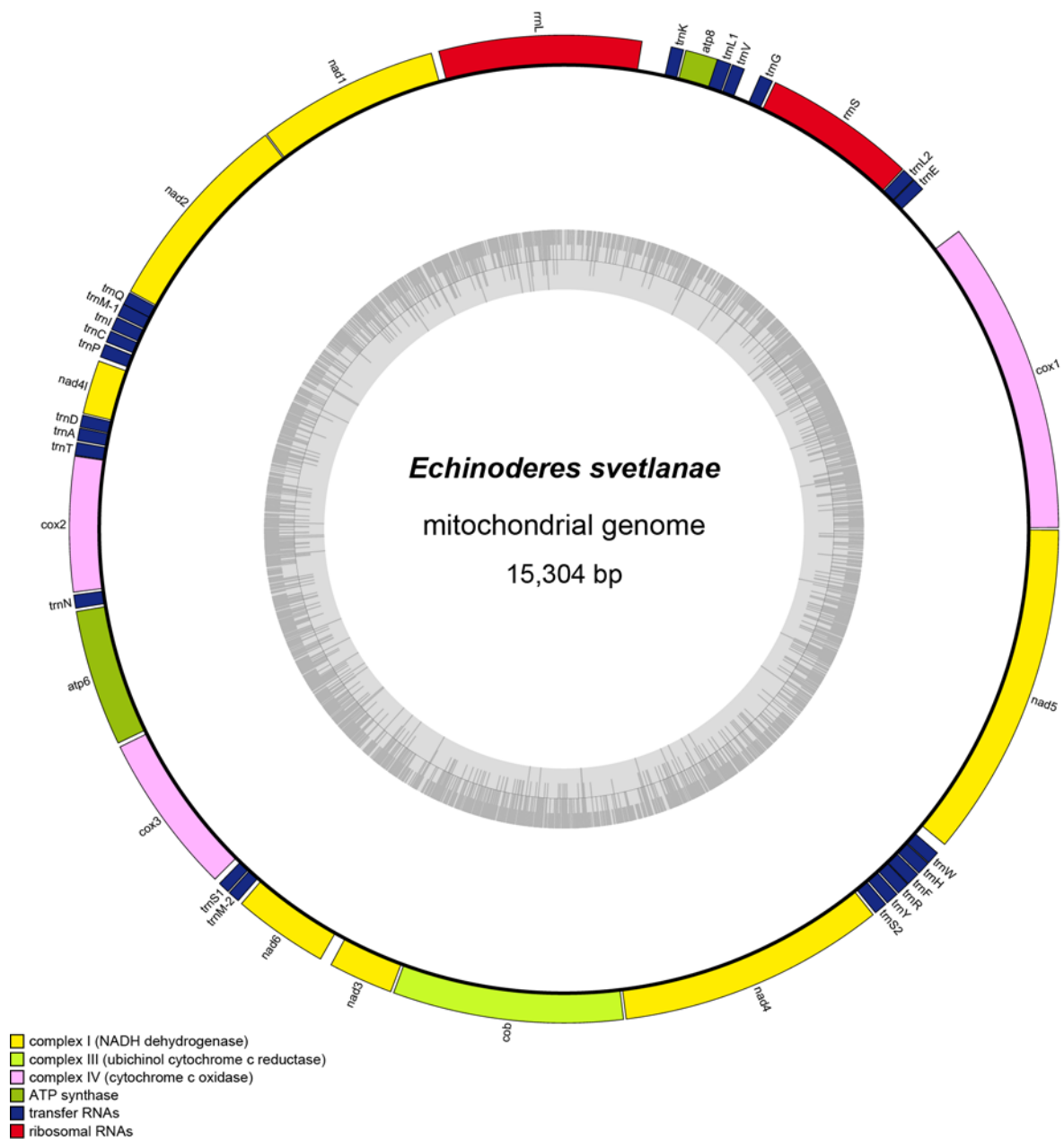

Supplement: S1 Fig — (PDF) [file pone.0165072.s001.pdf]

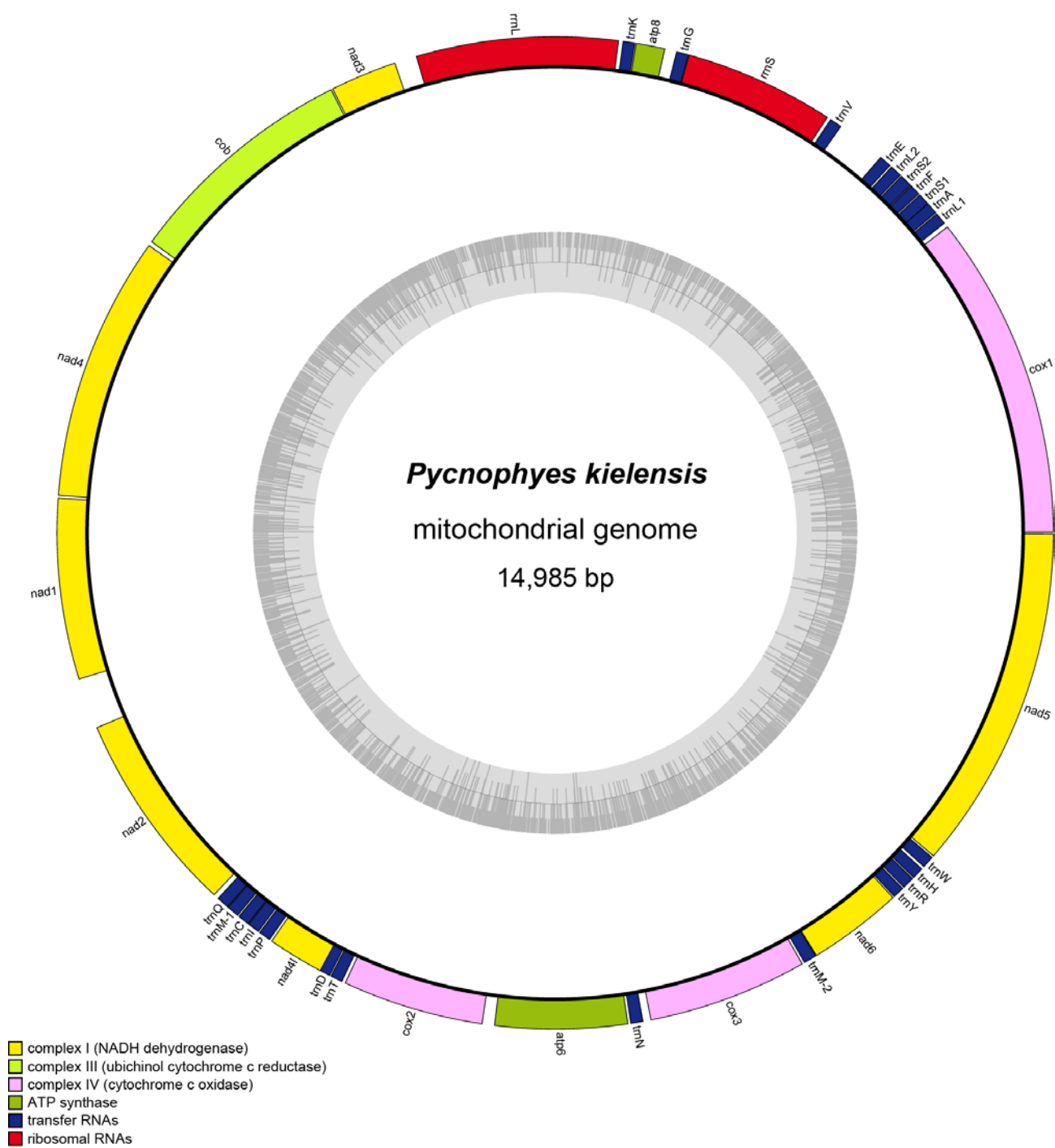

Supplement: S2 Fig — (PDF) [file pone.0165072.s002.pdf]

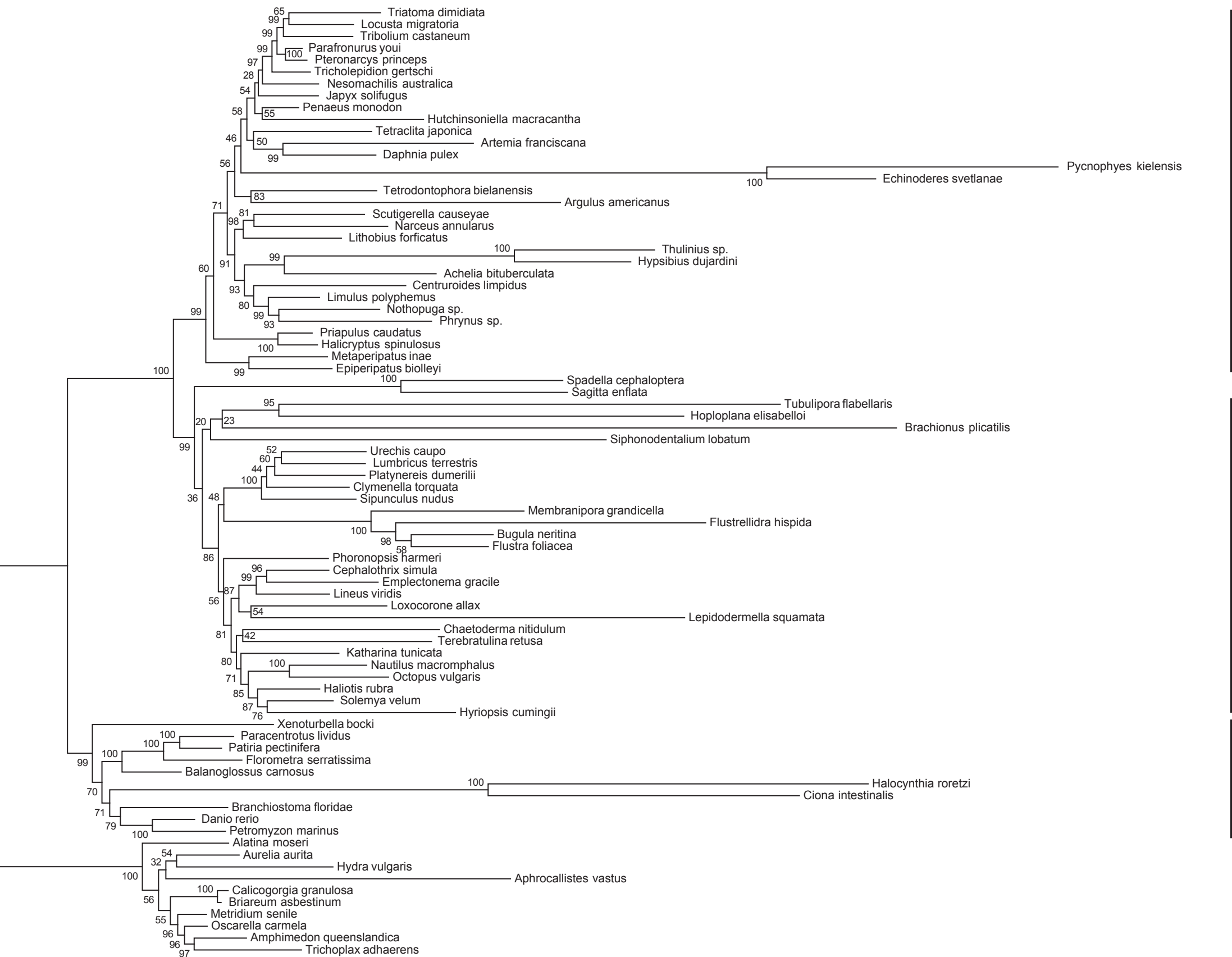

Ecdysozoa

Lophotrochozoa

Deuterostomia

0.5

Supplement: S5 Fig — Numbers at the branches indicate Bayesian posterior probabilities as percent. (PDF) [file pone.0165072.s005.pdf]

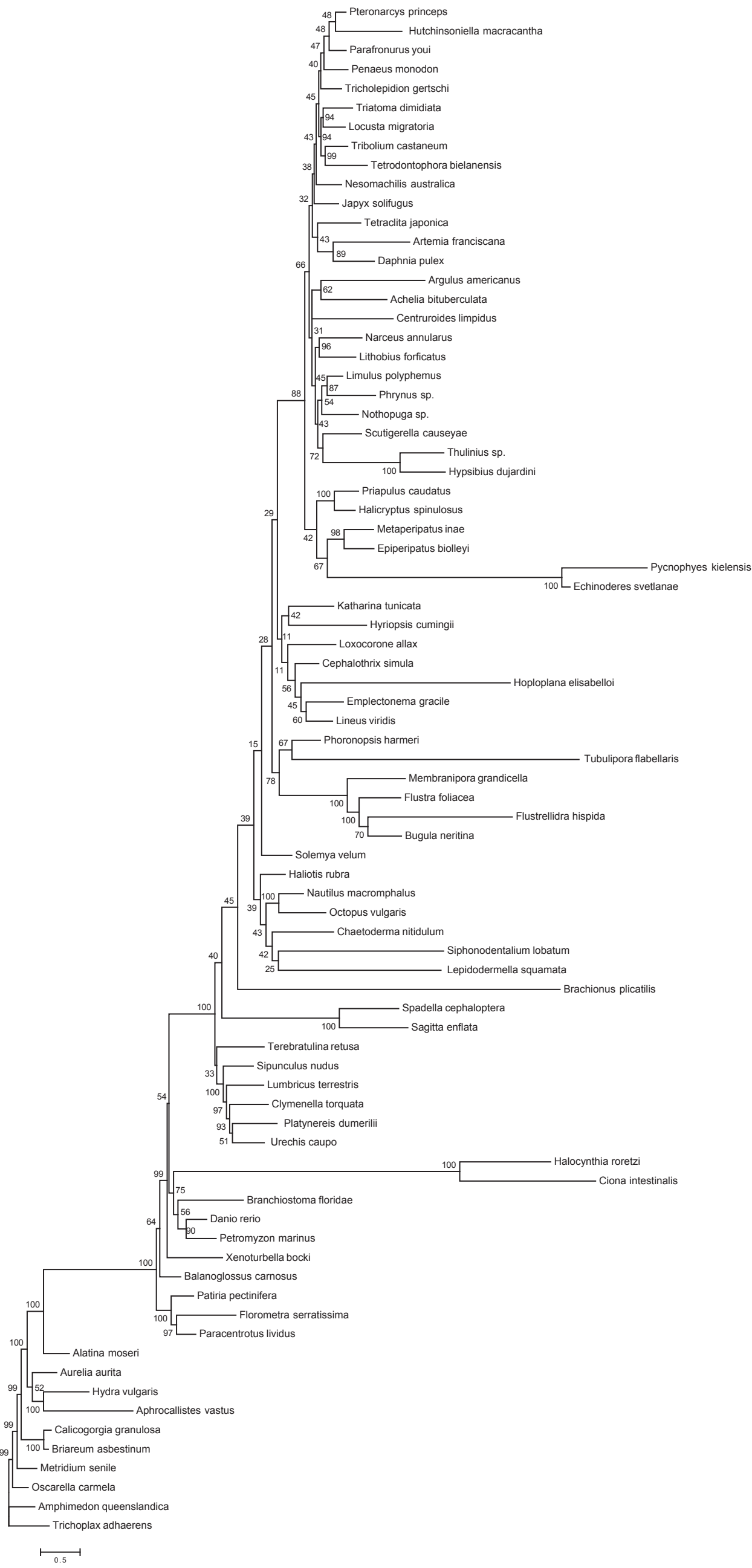

Ecdysozoa

Lophotrochozoa

Lophotrochozoa

Deuterostomia

Supplement: S6 Fig — Numbers at the branches indicate Bayesian posterior probabilities as percent. (PDF) [file pone.0165072.s006.pdf]

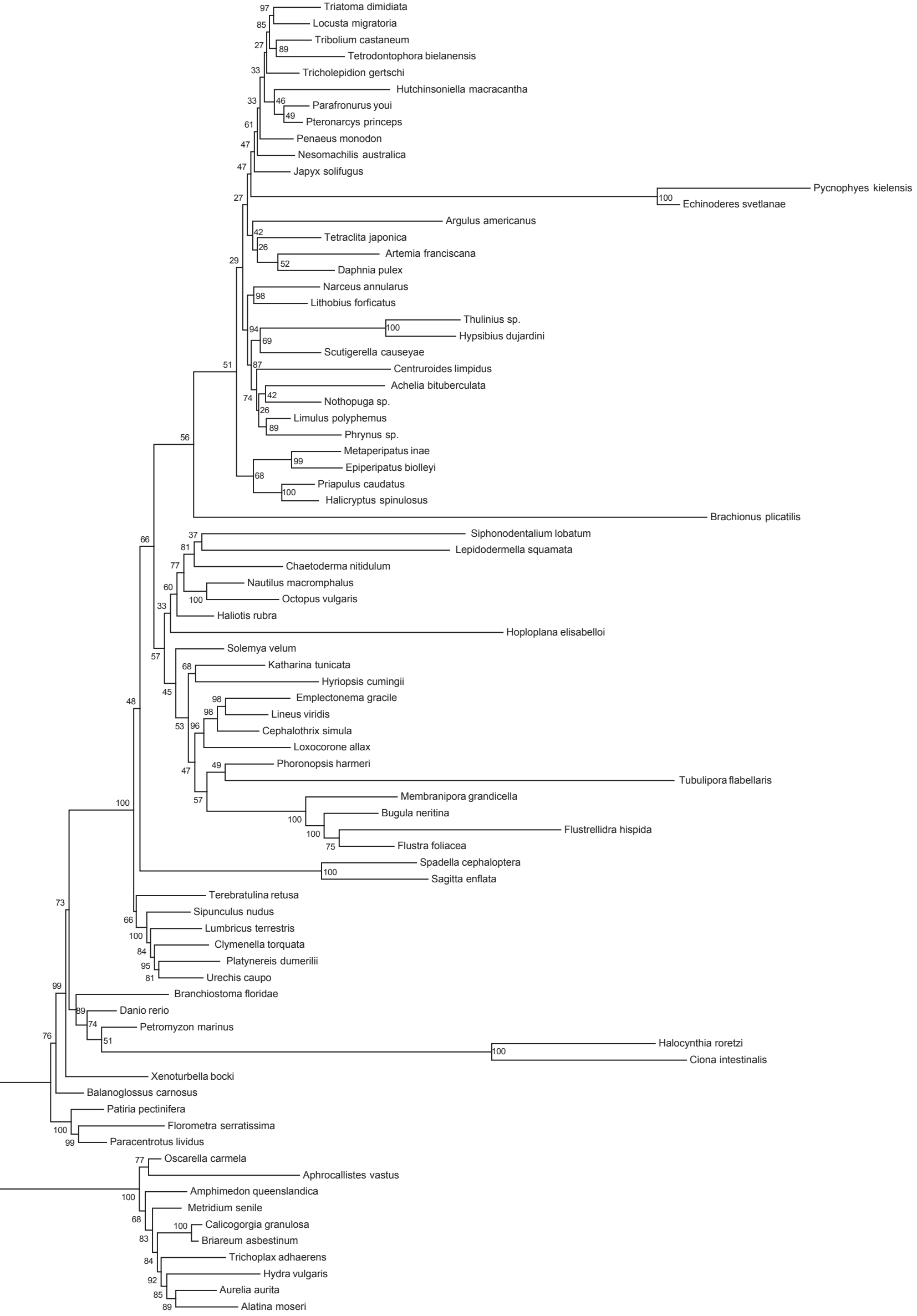

Ecdysozoa

Lophotrochozoa

Lophotrochozoa

Deuterostomia

Supplement: S7 Fig — Numbers at the branches indicate Bayesian posterior probabilities as percent values. (PDF) [file pone.0165072.s007.pdf]

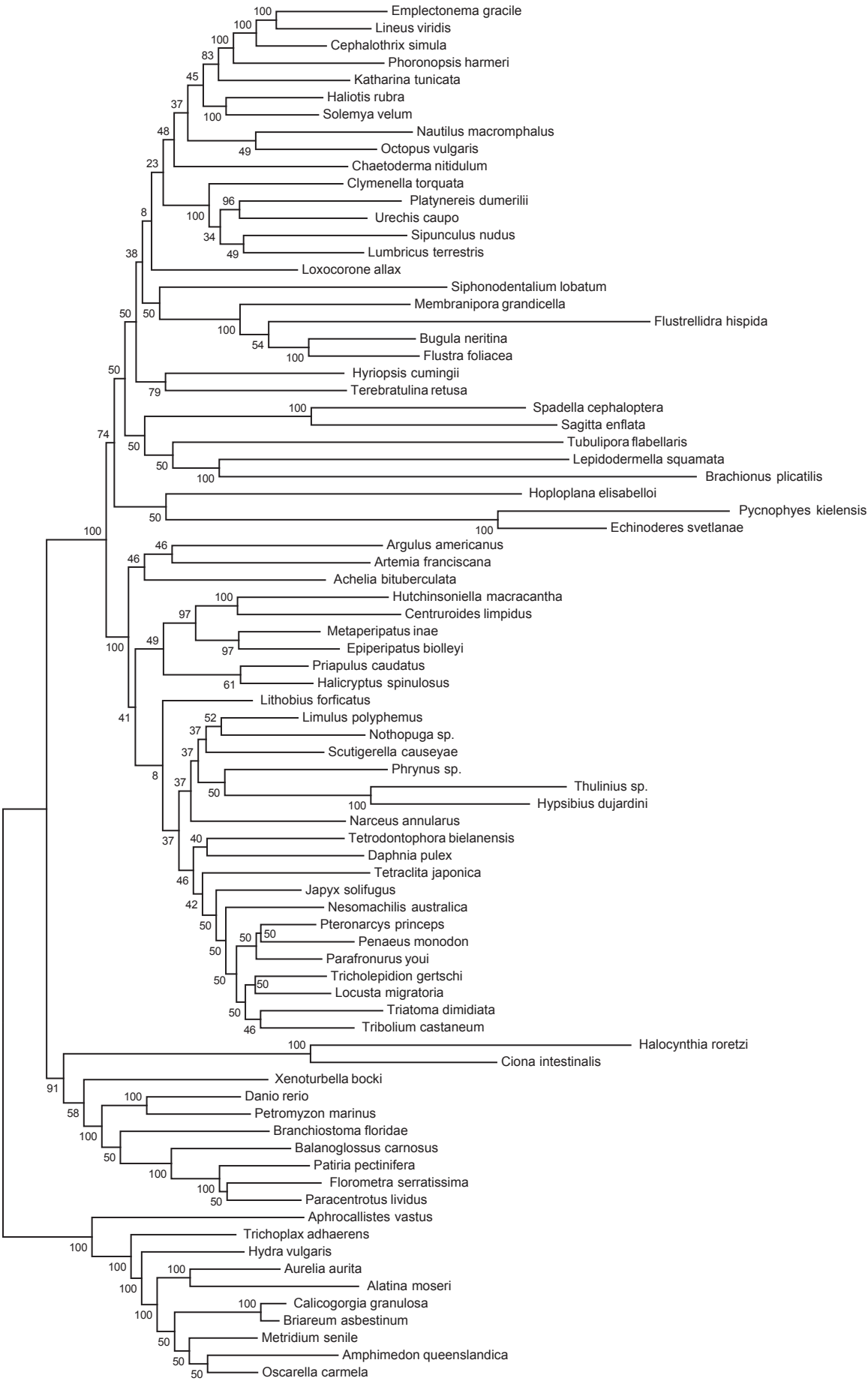

Lophotrochozoa

Lophotrochozoa

Ecdysozoa

Deuterostomia

Supplement: S8 Fig — Numbers at the branches indicate Bayesian posterior probabilities as percent values. (PDF) [file pone.0165072.s008.pdf]

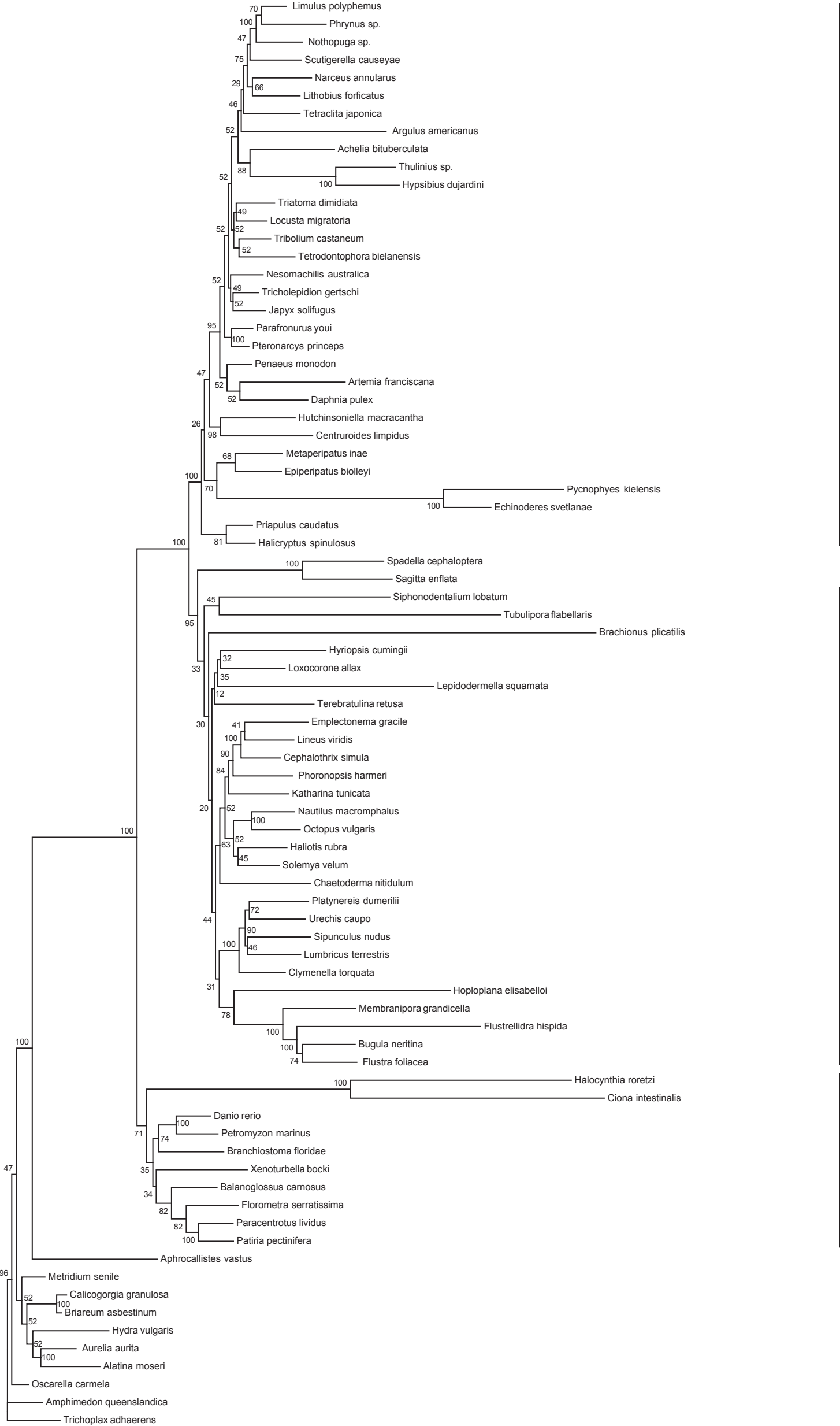

Ecdysozoa

Lophotrochozoa

Deuterostomia

Supplement: S9 Fig — Numbers at the branches indicate Bayesian posterior probabilities as percent values. (PDF) [file pone.0165072.s009.pdf]
