# Supplementary material for: Mitochondrial Genomes of Kinorhyncha: trnM Duplication and New Gene Orders within Animals
Source: PLoS One. 2016 Oct 18;11(10):e0165072. doi: 10.1371/journal.pone.0165072 (PMC5068742; doi:10.1371/journal.pone.0165072)
Supplement: S3 Fig — (PDF) [file pone.0165072.s003.pdf]

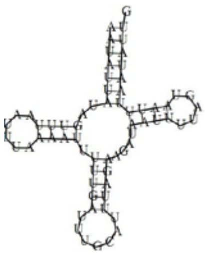

Alanine

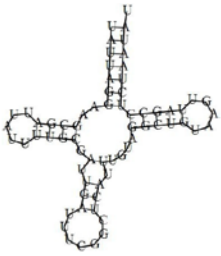

Arginine

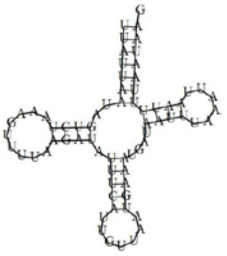

Asparagine

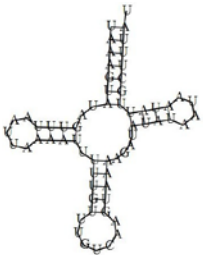

Aspartic Asid

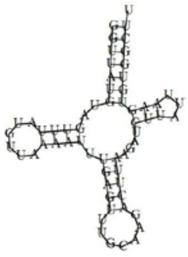

Cysteine

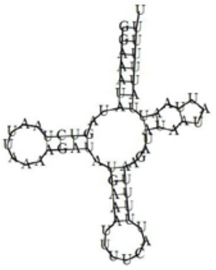

Glutamic Asid

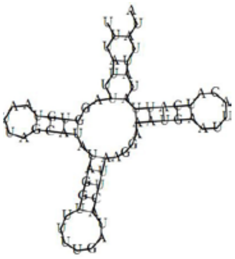

Glutamine

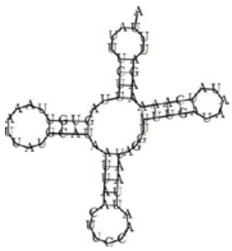

Glycine

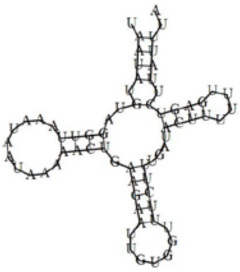

Histidine

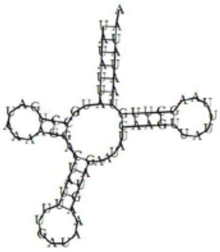

Isoleucine

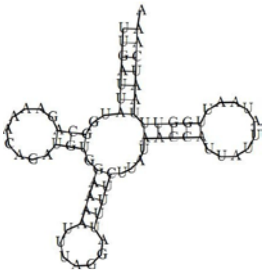

Leucine (UAG)

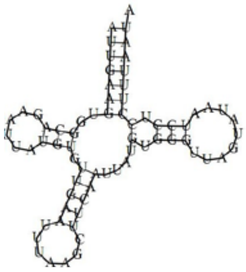

Leucine (UAA)

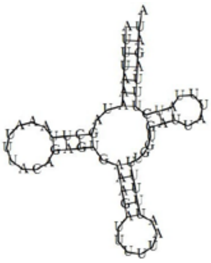

Lysine

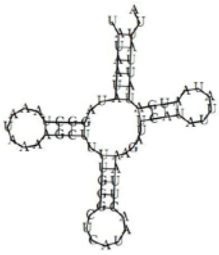

Methionine-1

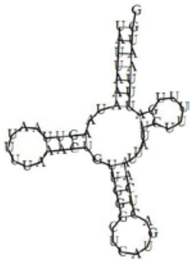

Methionine-2

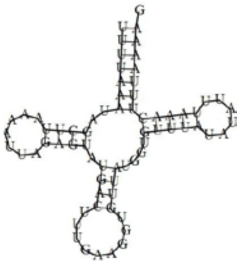

Phenylalanine

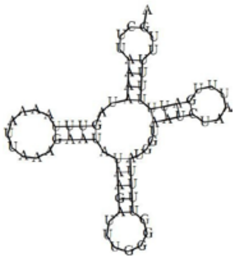

Proline

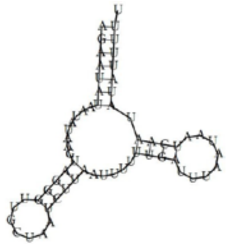

Serine (GCU)

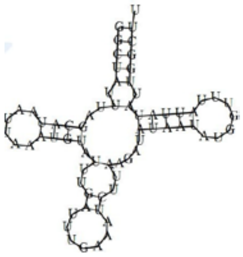

Serine (UGA)

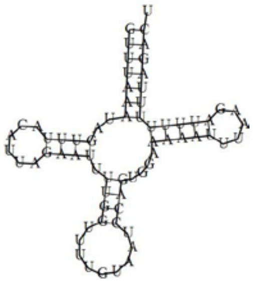

Threonine

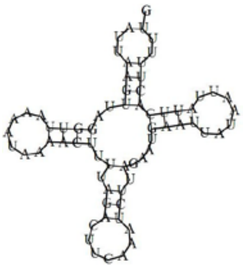

Tryptophan

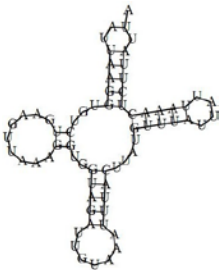

Tyrosine

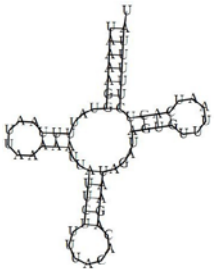

Valine
