# Supplementary material for: Mitochondrial Genomes of Kinorhyncha: trnM Duplication and New Gene Orders within Animals
Source: PLoS One. 2016 Oct 18;11(10):e0165072. doi: 10.1371/journal.pone.0165072 (PMC5068742; doi:10.1371/journal.pone.0165072)
Supplement: S1 Table — (DOC) [file pone.0165072.s010.doc]

| Species | Accession number | Species | Accession number |
| --- | --- | --- | --- |
| *Achelia bituberculata* | NC_009724 | *Locusta migratoria* | NC_001712 |
| *Alatina moseri* | NC_016988, NC_017008, NC_017009, NC_017010, NC_017011, NC_017012, NC_017013, NC_017014 | *Lumbricus terrestris* | NC_001673 |
| *Albinaria caerulea* | NC_001761 | *Membranipora grandicella* | NC_018355 |
| *Amphimedon queenslandica* | NC_008944 | *Metaperipatus inae* | NC_010961 |
| *Aphrocallistes vastus* | NC_010769 | *Metridium senile* | NC_000933 |
| *Artemia franciscana* | NC_001620 | *Narceus annularus* | NC_003343 |
| *Argulus americanus* | NC_005935 | *Nautilus macromphalus* | NC_007980 |
| *Aurelia aurita* | NC_008446 | *Nesomachilis australica* | NC_006895 |
| *Balanoglossus carnosus* | NC_001887 | *Nothopuga sp.* LP-2008 | NC_009984 |
| *Brachionus plicatilis* | NC_010472, NC_010484 | *Octopus vulgaris* | NC_006353 |
| *Branchiostoma floridae* | NC_000834 | *Oscarella carmela* | NC_009090 |
| *Briareum asbestinum* | NC_008073 | *Paracentrotus lividus* | NC_001572 |
| *Bugula neritina* | NC_010197 | *Parafronurus youi* | NC_011359 |
| *Calicogorgia granulosa* | NC_023345 | *Patiria pectinifera* | NC_001627 |
| *Centruroides limpides* | NC_006896 | *Penaeus monodon* | NC_002184 |
| *Cephalothrix simula* | NC_012821 | *Petromyzon marinus* | NC_001626 |
| *Ciona intestinalis* | NC_004447 | *Phoronopsis harmeri* | NC_018761 |
| *Chaetoderma nitidulum* | NC_013846 | *Phrynus sp.* 1 SEM-2008 | NC_000931 |
| *Clymenella torquata* | NC_006321 | *Platynereis dumerilii* | NC_000931 |
| *Danio rerio* | NC_002333 | *Priapulus caudatus* | NC_008557 |
| *Daphnia pulex* | NC_000844 | *Pteronarcys princeps* | NC_006133 |
| *Emplectonema gracile* | NC_016952 | *Sagitta enflata* | NC_013814 |
| *Epiperipatus biolleyi* | NC_009082 | *Scutigerella causeyae* | NC_008453 |
| *Florometra serratissima* | NC_001878 | *Siphonodentalium lobatum* | NC_005840 |
| *Flustra foliacea* | NC_016722 | *Sipunculus nudus* | NC_011826 |
| *Flustrellidra hispida* | NC_008192 | *Solemya velum* | NC_017612 |
| *Halicryptus spinulosus* | NC_020030 | *Spadella cephaloptera* | NC_006386 |
| *Haliotis rubra* | NC_005940 | *Terebratalina retusa* | NC_000941 |
| *Halocynthia roretzi* | NC_002177 | *Tetraclita japonica* | NC_008974 |
| *Holoplana elisabelloi* | NC_028200 | *Tetrodontophora bielanensis* | NC_002735 |
| *Hutchinsoniella macracantha* | NC_005937 | *Thulinius sp.* DVL-2010 | NC_015829 |
| *Hydra vulgaris* | NC_011220, NC_011221 | *Triatoma dimidiata* | NC_002609 |
| *Hypsibius dujardini* | NC_014848 | *Tribolium castaneum* | NC_003081 |
| *Hyriopsis cumingii* | NC_011763 | *Tricholepidion gertschi* | NC_005437 |
| *Japyx solifugus* | NC_007214 | *Trichoplax adhaerens* | NC_008151 |
| *Katharina tunicata* | NC_001636 | *Tubulipora flabellaris* | NC_015646 |
| *Lepidodermella squamata* | NC_026985 | *Urechis caupo* | NC_006379 |
| *Lineus viridis* | NC_012889 | *Vargula hitgendorfii* | NC_005306 |
| *Lithobius forficatus* | NC_002629 | *Xenoturbella bocki* | NC_008556 |
| *Loxocorone allax* | NC_010431 |  |  |
