# Supplementary material for: Mitochondrial Genomes of Kinorhyncha: trnM Duplication and New Gene Orders within Animals
Source: PLoS One. 2016 Oct 18;11(10):e0165072. doi: 10.1371/journal.pone.0165072 (PMC5068742; doi:10.1371/journal.pone.0165072)
Supplement: S2 Table — (DOC) [file pone.0165072.s011.doc]

| Codon | Number of codons | | Invertebrate  mitochondrial code | GenDecoder results* | | FACIL results** | |
| --- | --- | --- | --- | --- | --- | --- | --- |
|  | *E. svetlanae* | *P. kielensis* |  | *E.*  *svetlanae* | *P. kielensis* | *E.*  *svetlanae* | *P.*  *kielensis* |
| TGC | 2 | 3 | C | P (0,89) | A (0,77) | L (0,01) | A (0,22) |
| ACG | 4 | 3 | T | T (0,5) | S (0,98) | T (0,99) | S (0,05) |
| ATG | 55 | 57 | M | L (0,35) | M (0,35) | L (0,93) | L (0,49) |
| ATA | 185 | 283 | M | M (0,39) | M (0,27) | L (0,14) | L (0,43) |
| TGT | 66 | 34 | C | C (0,27) | C (0,25) | A (0,63) | C (0,38) |
| TTC | 5 | 18 | F | F (0,56) | F (0,6) | L (0,78) | F (1) |
| ATC | 4 | 12 | I | no data | I (0,5) | no data | L (0,7) |

Differences in kinorhynchs mitogenomes from the invertebrate mitochondrial code predicted by GenDecoder and FACIL.

* - the number in brackets shows the frequent of amino acid in conservative sites of alignment.

** - the number in brackets shows the percentage of RF3 (Random forest 3 set) trees supporting the predicted translation.
